# Hepatitis C Virus Is a Weak Inducer of Interferon Alpha in Plasmacytoid Dendritic Cells in Comparison with Influenza and Human Herpesvirus Type-1

**DOI:** 10.1371/journal.pone.0004319

**Published:** 2009-02-02

**Authors:** Françoise Gondois-Rey, Clélia Dental, Philippe Halfon, Thomas F. Baumert, Daniel Olive, Ivan Hirsch

**Affiliations:** 1 Institut National de la Santé et de la Recherche Médicale (INSERM) UMR891, Centre de Recherche en Cancérologie de Marseille, Marseille, France; 2 Institut Paoli-Calmettes, Marseille, France; 3 Université Méditerranée, Marseille, France; 4 Department of Virology, Alphabio Laboratory, Marseille, France; 5 INSERM U748, Université Louis Pasteur, Strasbourg, France; Karolinska Institutet, Sweden

## Abstract

Plasmacytoid dendritic cells (pDCs) are responsible for the production of type I IFN during viral infection. Viral elimination by IFN-α-based therapy in more than 50% of patients chronically infected with hepatitis C virus (HCV) suggests a possible impairment of production of endogenous IFN-α by pDCs in infected individuals. In this study, we investigated the impact of HCV on pDC function. We show that exposure of pDCs to patient serum- and cell culture-derived HCV resulted in production of IFN-α by pDCs isolated from some donors, although this production was significantly lower than that induced by influenza and human herpesvirus type 1 (HHV-1). Using specific inhibitors we demonstrate that endocytosis and endosomal acidification were required for IFN-α production by pDCs in response to cell culture-derived HCV. HCV and noninfectious HCV-like particles inhibited pDC-associated production of IFN-α stimulated with Toll-like receptor 9 (TLR9) agonists (CpG-A or HHV-1) but not that of IFN-α stimulated with TLR7 agonists (resiquimod or influenza virus). The blockade of TLR9-mediated production of IFN-α, effective only when pDCs were exposed to virus prior to or shortly after CpG-A stimulation, was already detectable at the IFN-α transcription level 2 h after stimulation with CpG-A and correlated with down-regulation of the transcription factor IRF7 expression and of TLR9 expression. In conclusion, rapidly and early occurring particle–host cell protein interaction during particle internalization and endocytosis followed by blockade of TLR9 function could result in less efficient sensing of HCV RNA by TLR7, with impaired production of IFN-α. This finding is important for our understanding of HCV-DC interaction and immunopathogenesis of HCV infection.

## Introduction

Plasmacytoid dendritic cells (pDCs) are a highly specialized subset of dendritic cells that function as sentinels for viral infection and are responsible for production of large amounts of type I IFN during viral infection [1]–[3]. pDCs are able to detect genetic material of virus particles after their degradation in endosomal compartments *via* interaction with Toll-like receptors (TLR) [4]. pDCs are able to detect DNA of inactivated human herpesvirus types 1 (HHV-1) and 2 (HHV-2) *via* TLR9 (AAQ89443) [5], [6], and they are able to detect single-stranded RNA of inactivated influenza virus and of HIV-1 *via* TLR7 (AAQ88659) [7]–[10]. However, inactivation renders some single-stranded RNA viruses, like measles [11], respiratory syncytial virus [11], [12], and vesicular stomatitis virus [13], incapable of inducing potent pDC-associated production of IFN-α. The recognition of such viruses by TLR7 and the production of IFN-α (NP 076918) by pDCs require transport of cytosolic viral replication intermediates into lysosomes by the process of autophagy [13]. Recent results show that replicating HCV induces an autophagic response in immortalized human hepatocytes [14].

The eradication of hepatitis C virus (HCV) in more than 50% of chronically infected patients by treatment with IFN-α in combination with ribavirin [15], [16] suggests that pDCs can play a major role in the control of HCV infection. Several studies that analyzed the function of pDCs in chronically infected patients compared with those from normal subjects reported a markedly reduced IFN-α production after *ex vivo* exposure of pDCs to agonists of TLR9 (A/D type CpG oligonucleotides) and TLR7 (imidazoquinoline components, *e.g.,* R848, resiquimod) [17]–[20]. However, other reports found no difference between these groups [21], [22]. Whereas in these studies, the pDCs obtained from patients with chronic HCV infection were exposed to synthetic stimulators of TLR7 or TLR9 in the absence of HCV, more recent studies investigated the effects of TLR7 or TLR9 ligands on pDCs purified from healthy donors in the presence of cell culture-prepared HCV (HCVcc) [21], [23]. These reports have shown that exposure of pDCs from healthy donors to HCVcc is not followed by expression of the HCVcc genome and viral replication, that HCVcc does not induce pDC-associated production of IFN-α and cell differentiation [21], [23], and that, in addition, HCVcc blocks IFN-α production mediated *via* TLR9 [23].

In contrast to these earlier studies, we show here that exposure of pDCs to HCV results in production of IFN-α by pDCs isolated from some donors, although this production is significantly lower than that induced by influenza and human herpesvirus type 1 (HHV-1). Production of IFN-α was sensitive to specific inhibitors of endocytosis and endosomal acidification and was resistant to virus inactivation. In order to better understand the mechanism of poor induction of IFN-α by HCVcc-exposed pDCs, we also studied the inhibition of TLR9-mediated IFN-α production with HCVcc [23] and with HCV-like particles (HCV-LPs) [24]–[26]. We conclude that the interaction of the viral particle with host cell factors during viral internalization and endocytosis followed by blockage of TLR9 signaling could result in less efficient sensing of HCV RNA by TLR7, with impaired production of IFN-α. On the basis of these results we propose a new mechanism by which HCV can evade recognition by pDCs.

## Results

### HCV does not induce maturation of purified pDCs

**Figure 1:**
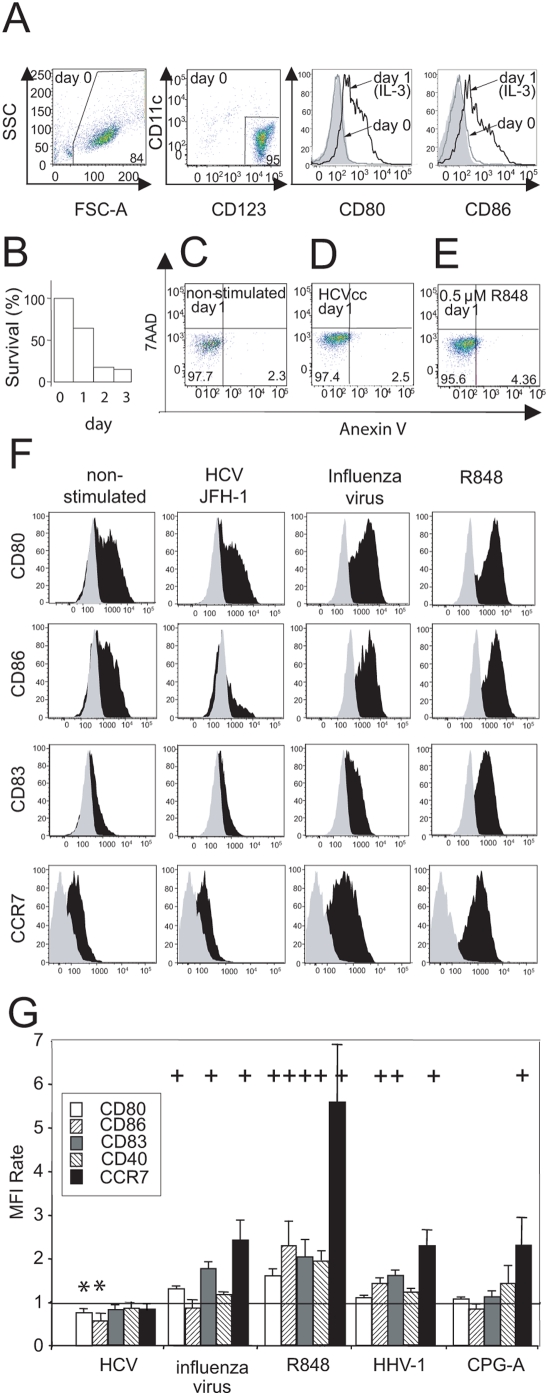
Expression of differentiation markers of pDCs exposed to HCVcc. (A) Cell cultures of pDCs were purified from PBMCs by use of magnetic beads. The purity of the isolated pDCs was determined from flow cytometry analysis after staining with CD123 and CD11c. Numbers displayed are percentages of positive cells. Levels of purity from 75% to 95% were repeatedly obtained. Cell cultures of pDCs were adjusted to a concentration of 10^6^ cells/ml in the presence of IL-3, and the expression of the cell surface markers CD80 and CD86 was determined immediately after cell separation (day 0, gray line) or after 1-day culture (day 1, black line). Isotype control is shown by the gray shadowed area. (B) pDC survival in the presence of IL-3. (C–E) HCVcc does not induce pDCs apoptosis. (C) Cell cultures of pDCs in the presence of IL-3 were (D) inoculated at a multiplicity of 100 genome-containing virus particles of HCVcc per cell or (E) stimulated with 0.5-µM resiquimod (R848) in a total volume of 200 µl. pDCs were stained with Annexin V and 7ADD 1 day post-stimulation. (F) pDCs were inoculated at a multiplicity of 100 virus genome copies of HCVcc or influenza virus A/H3N2/Johannesburg, or they were stimulated with 0.5-µM resiquimod (R848) in a total volume of 200 µl. Expression of CD80, CD86, CD83, and CCR7 was determined in CD123^+^HLA-DR^+^ gated live cells 1 day post-stimulation. Black area, virus- or R848-exposed pDCs; gray area, isotype control. Data are representative of 10 independent experiments with pDCs of different donors that gave comparable results. (G) The mean fluorescence intensity (MFI) rate corresponds to the MFI of a sample divided by that of non-stimulated cells. The data show means and SEM of 10 independent experiments with pDCs of different donors stimulated with HCVcc, influenza virus A/H3N2/Johannesburg, R848, HHV-1 KOS, or CpG-A. +, significant induction of the respective cell surface marker as compared with the corresponding non-stimulated control; *, significant suppression of the respective cell surface marker as compared with the corresponding non-stimulated control (non-parametric Mann-Whitney test).

First, we compared the capacity of molecular clone HCVcc JFH-1 to induce pDC differentiation with the capacities of resiquimod, influenza virus A/H3N2/Johannesburg, and HHV-1 KOS (Figure 1). Since TLR7 recognizes and is activated by viral RNA [27], [28], and TLR9 by viral DNA [5], [29], we normalized the quantity of assayed viruses on the basis of the number of virus genome copies as determined with PCR (not shown). Purified pDCs from normal healthy donors (Figure 1A) were inoculated with examined viruses at a multiplicity of 100 genome copies per cell. All viruses were purified by ultracentrifugation through a cushion of 20% sucrose to minimize the presence of bystander activation factors. Analysis of the purity of concentrated stocks of HCVcc by electron microscopy showed the presence of particles of 60–80 nm in diameter and the absence of detectable amounts of cellular DNA and RNA (supplementary Figure S1).

One-day-culture of pDCs in medium supplemented with IL-3, an important factor for pDC survival, resulted in partial pDC maturation (Figure 1A). pDC viability declined with a half-time of approximately 1 day (Figure 1B). Flow cytometry analysis of Annexin V/7ADD-stained cells revealed no increase in the proportion of apoptotic cells among pDCs inoculated with HCVcc in comparison with non-stimulated or resiquimod-stimulated pDCs (Figure 1C–E). Inoculation of pDCs from 10 healthy donors with HCVcc slightly but significantly down-regulated the expression of CD80 (ABK41933) and CD86 (CAG46642), whereas the expression of other assayed surface markers (CD40 (AAL92924), CD83 (CAB63843), and CCR7 (NP 001829)) remained unchanged in comparison with culture without stimulation (Figure 1F and 1G). Stimulation of pDCs with TLR7 agonist resiquimod strongly increased the expression of CD40, CD80, CD83, CD86, and CCR7. In contrast, stimulation of pDC with TLR9 agonist CpG-A resulted in significantly increased expression of CCR7 and in only a moderate increase of the expression of CD80, CD83, and CD40 (Figure 1G). Stimulation with the natural agonist of TLR7 influenza virus significantly increased the expression of CD80, CD83, and CCR7, whereas stimulation with the natural agonist of TLR9 HHV-1 significantly increased the expression of CD86, CD83, and CCR7. Taken together, these findings show that HCV did not induce, but rather blocked, the maturation of purified pDCs and had no effect on their apoptosis.

### IFN-α production induced by cell culture- and patient-derived HCV in pDCs

**Figure 2:**
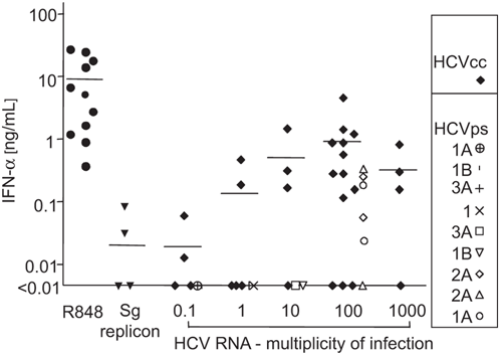
Secretion of IFN-α induced with cell culture-derived HCVcc and patient-derived HCV in pDCs from different normal healthy donors. Purified pDCs were inoculated with different quantities of HCVcc (genotype 2) or patient-derived HCV (genotypes 1a, 1b, 2a, and 3a). Viral genomes were quantified by semiquantitative RT-PCR. As negative control, a suspension prepared from cell-free supernatant of Huh7.5 cells transfected with HCV subgenomic (sg) replicon was used. This negative control suspension was concentrated and purified in the same manner as the viral suspension used for exposure of pDCs to 100 genome-containing virus particles per cell. Resiquimod (R848, 0.5 µM) was used as a positive control. Cell cultures of pDCs purified from different normal healthy donors adjusted to a concentration of 10^6^ cells/ml in the presence of IL-3 were inoculated with HCV (quantified by HCV RNA copies) in a total volume of 200 µl. Secretion of IFN-α in cell-free supernatant was determined by ELISA 1 day post-stimulation. Identical symbols at a given multiplicity of infection indicate results with pDCs of different donors.

Next, we assayed IFN-α production induced by increasing doses of infectious HCVcc in purified pDCs from a small panel of donors (Figure 2). pDCs of 13 donors incubated with HCV JFH preparations at a dose of 100 HCV RNA copies per target cell secreted 1.27±0.39 (mean±SEM) ng of IFN-α per milliliter. pDCs of three donors did not produce detectable amounts of IFN-α (the detection level in ELISA was 10 pg of IFN-α per milliliter), whereas pDCs from other three donors consistently produced more than 1 ng of IFN-α per milliliter.

pDCs exposed to 0.5 µM resiquimod, used as a positive control, produced 9.2±4.1 ng of IFN-α per milliliter. IFN-α levels produced by pDCs stimulated with HCVcc did not show any correlation with levels of IFN-α induced by resiquimod (supplementary Figure S2). The three donors with absent IFN-α production after stimulation with HCVcc (Figure 2), produced significant levels of IFN-α (730, 5,352 and 5,827 pg per milliliter) after stimulation with resiquimod (supplementary Figure S2). In addition to pDCs, myeloid DCs and macrophages respond to CpG-DNA and produce small amounts of type-I IFN. In our experiments, magnetic bead-purified myeloid DCs, the principal source of possible contamination of pDCs, did not produce any IFN-α after exposure to HCV. In addition to HCVcc, we incubated pDCs with a control suspension prepared in the same manner as the viral stock from cell-free supernatant of Huh7.5 cells transfected with HCV H/SG-neo (L+I) subgenomic replicon [30]. Because Huh7.5.1 cells were apoptotic at the time of HCVcc harvest, we rendered the control culture apoptotic by UV-irradiation [23]. pDCs from four donors exposed to this control HCVcc virion-free supernatant did not produce significant quantities of IFN-α (Figure 2) confirming that pDC-associated production of IFN-α was induced by HCV and not by cellular components.

To test the biological relevance of results obtained with HCVcc (genotype 2), we exposed pDCs to similar doses of patient-derived HCV (genotypes 1a, 1b, 2a, and 3a; Figure 2) compared with HCVcc. pDC-associated production of IFN-α increased with increasing levels of HCV genome copies, although at a lower rate as observed for HCV JFH-1 CAI41940). Taken together, these data suggest that exposure of pDCs to cell culture- or patient-derived HCV resulted in production of IFN-α, although it showed a high degree of variability among pDCs from different donors.

### In contrast to influenza virus and HHV-1, HCV is a weak inducer of pDC-associated production of IFN-α and TNF-α

**Figure 3:**
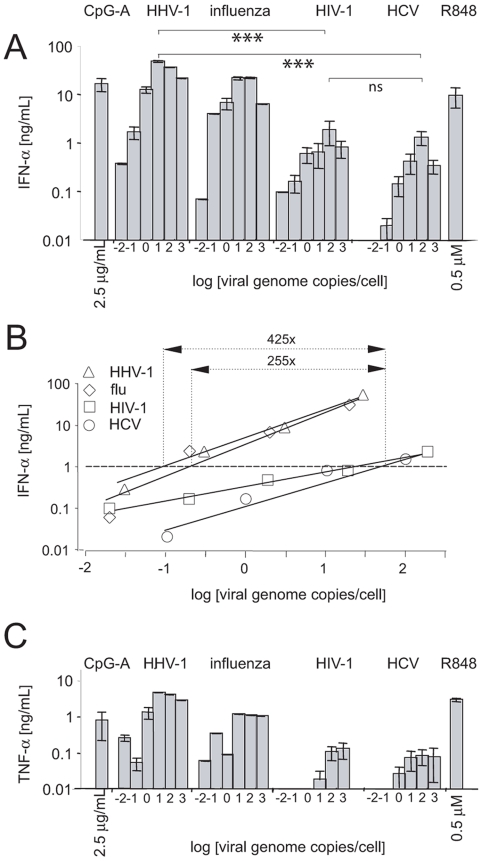
Secretion of IFN-α and TNF-α by purified pDCs inoculated with HCVcc, HIV-1 NL4.3, influenza virus A/H3N2/Johannesburg, or HHV-1 KOS, or stimulated with resiquimod or CpG-A. Cell cultures of pDCs purified from PBMCs of different healthy donors (*n*_HCV_ = 13, *n*_HIV-1_ = 8, *n*_HHV-1_ = 8, *n*_FLU_ = 3) adjusted to a concentration of 10^6^ cells/ml in the presence of IL-3 were inoculated with increasing doses of examined viruses in a total volume of 200 µl. The quantity of virus particles was determined by means of semiquantitative RT-PCR for HCV, HIV, and influenza virus, and by means of semiquantitative PCR for HHV-1. (A) Secretion of IFN-α. For HCV, the same data as in Figure 2 are shown. (B) Capacity of HCV, HHV-1, or influenza virus to stimulate IFN-α production as a function of the number of virus genome copies per cell. Analysis of linear parts of the activation curves shown in panel A was performed by means of linear regression. (C) Secretion of TNF-α. Secretion of IFN-α and TNF-α in cell-free supernatant was determined by means of ELISA analysis 1 day post-stimulation. ***, statistically significant differences (*p*<0.0001, Mann-Whitney test); R848, resiquimod.

Next, we compared the capacity of HCV JFH-1 to induce pDC-associated secretion of IFN-α and TNF-α (CAI41940) with the capacities of HIV-1 LAI, influenza virus A/H3N2/Johannesburg, and HHV-1 KOS. pDC-associated production of IFN-α in different donors (*n*_HCV_ = 13, *n*_HIV-1_ = 8, *n*_HHV-1_ = 8, *n*_FLU_ = 3) increased with levels of viral genome copies and reached a maximum at a dose of 100 genome copies per cell for HCVcc, HIV-1, and influenza virus, whereas the optimum for HHV-1 was reached at 10 genome-containing virus particles per cell (Figure 3A). Regression analysis of the linear parts of the activation curves showed that approximately 0.12 (HHV-1), 0.2 (influenza virus), 38 (HIV-1), or 51 (HCVcc) genome copies per cell were necessary for the production of 1 ng of IFN-α per milliliter (Figure 3B). Comparing the viral genome copy number inducing the same quantity of IFN-α production, HHV-1 and influenza virus are 425-fold and 255-fold more potent than HCVcc in inducing IFN-α production. The difference between the capacity of HCVcc and HIV-1 to induce pDC-associated production of IFN-α was not significant (Figure 3A).

Complementing the investigation of IFN-α production, we studied the stimulatory effect of the four viruses on pDC-associated TNF-α production (Figure 3C). Similar as seen for IFN-α, incubation of pDCs with HCV JFH-1 resulted in a dose-dependent production of TNF-α, albeit the level of TNF-α was markedly lower than the level of TNF-α induced by influenza virus and HHV-1. Thus, we conclude that HCV, compared to influenza virus and HHV-1, is a weak inducer of pDC-associated production of IFN-α and TNF-α.

### Activation of pDCs by HCV occurs during viral endocytosis

To investigate whether endocytosis was required for recognition of HCV, we tested several inhibitors that block the cellular uptake of particular structures, including viruses (Table 1). Dimethyl amiloride, cytochalasin D, and chlorpromazine fully inhibited IFN-α secretion, suggesting that viral endocytosis is required for pDCs stimulation. Incubation of pDCs with chloroquine, quinacrine, and bafilomycin A1 resulted in complete inhibition of HCVcc-induced IFN-α secretion, showing that acidification/maturation of the endosomes is necessary for activation of pDCs. Although less effective than chloroquine, quinacrine or BafA1, NH_4_Cl inhibited HCVcc-induced IFN-α secretion in a dose-dependent manner. Consistent with previous observations that activation of TLR7 occurs within endosomes, inhibition of IFN-α production by inhibitors of endocytosis was also observed in response to resiquimod [31]. In conclusion, our findings suggest that endocytosis of the HCV particle, with subsequent acidification of endosomes, is required for pDC activation.

**Table 1:** Sensitivity of IFN-α production to inhibitors of endocytosis.

Endocytosis inhibitor	IFN-α production, %*	
	HCVcc	0.5 µM resiquimod
Dimethyl amiloride (50 µM)	<5%	<5%
Cytochalasin D (10 µM)	<5%	<5%
Chlorpromazine (6.25 µM)	<5%	<5%
Chloroquine (5 µM)	<5%	<5%
Quinacrine (5 µM)	<5%	<5%
NH_4_Cl (1 mM)	80%	45%
NH_4_Cl (10 mM)	<5%	<5%
Bafilomycin A1 (50 nM)	<5%	<5%

* Percentage of mock-treated control. pDCs (300,000 cells) were exposed to 3×10^7^ HCVcc RNA copies in a final volume 300 µl for 16 h.

### Inactivated HCV stimulates similar levels of pDC-associated production of IFN-α as replication-competent infectious virus

The majority of viruses that are presented to TLR by endocytosis has a similar IFN-α stimulatory potential whether they are exposed to pDCs in replication-competent or inactivated form [5]–[7], [9]. Exposure of pDCs to inactivated virus lead to reduction of IFN-α secretion by only 8% (thermo-inactivated virus) or 18% (UV-inactivated virus), whereas the infectious titers (tissue culture infectious dose) of HCVcc determined in Huh7.5.1 producer cells (TCID_Huh7.5.1_/ml) dropped 10,000-fold or 1,000-fold, respectively (Table 2). Similar levels of pDC-associated production of IFN-α stimulated with inactivated as with replication-competent infectious virus indicate that, HCV does not need to be replication-competent to induce IFN-α production in pDCs and is compatible with a major role of endocytosis in pDC activation.

**Table 2:** Exposure of pDCs to heat-inactivated and UV-inactivated virus stimulates production of IFN-α*.

	TCID_Huh7.5.1_/ml	TCID_Huh7.5.1_/ml	IFN-α	IFN-α
		%	pg/ml	%
HCV	10^5^	100	120±38	100
HCV, 30 min 56°C	<10^1^	<0.01	111±25	92
HCV	10^5^	100	175±42	100
HCV, UV (0.2 J/cm^2^)	10^2^	0.1	142±40	81

* 10^8^ HCVcc RNA copies and 10^6^ pDCs per milliliter.

### Exposure of pDCs to HCVcc prior to or shortly after CpG-A stimulation blocks TLR9-mediated production of IFN-α

**Figure 4:**
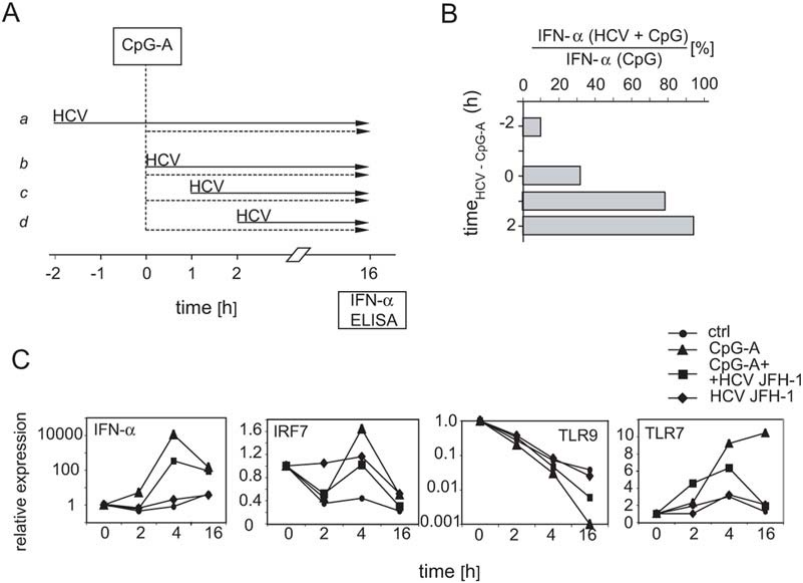
Kinetics of disruption of CpG-A-mediated stimulation of pDCs with HCVcc. (A) Flow chart protocol showing inoculation with HCV (solid arrows), treatment with CpG-A (dashed arrows), and IFN-α assay determined by ELISA. (B) Percentage of IFN-α secreted by pDCs that were stimulated with CpG-A and HCV relative to IFN-α secretion by pDCs stimulated only with CpG-A. Purified pDCs were inoculated with 100 HCVcc RNA copies per cell. (C) Effect of HCVcc on the expression of IFN-α, IRF-7, TLR7 and TLR9 mRNA. pDCs were primed with HCVcc and stimulated with CpG-A as shown in panel A, line *a*. The gene expression levels were determined with real-time PCR were normalized to GAPDH expression. Data are presented as fold induction over medium control at time zero (given the value of 1.0) and are from one of three representative experiments.

To investigate further the impact of HCV on pDC function, we studied kinetics of disruption of TLR9-mediated production of IFN-α. For this reason, we exposed the cells before or after stimulation with CpG-A to HCVcc, as shown in the chart flow protocol (Figure 4A). Priming with HCVcc 2 h before CpG-A stimulation (Figure 4A, line *a*) reduced the CpG-A-induced IFN-α levels by >90% (Figure 4B). Less pronounced (69%) inhibition of TLR9-mediated activation by HCVcc was observed when pDCs were exposed to HCVcc and CpG-A concomitantly (Figure 4A, line *b* and 4B). The inhibitory effect of HCVcc almost disappeared (12% inhibition) when the virus was added 1 h after CpG-A (Figure 4A, line *c*, and 4B). Thus, HCVcc inhibits TLR9-mediated production of IFN-α only when inoculation with HCVcc precedes or follows shortly after CpG-A stimulation.

To study events that precede secretion of IFN-α, we examined the expression of IFN-α, IRF7, TLR7, and TLR9 genes in pDCs that had been stimulated with CpG-A, with HCV JFH-1, or with both HCV JFH-1 and CpG-A (Figure 4A, line *a*, and 4C). In four experiments with pDCs purified from three donors, CpG up-regulated IFN-α expression in pDCs from 2,100- to 12,000-fold by 4 h, while simultaneous stimulation with HCVcc reduced the up-regulation of IFN-α expression by 18 to 76 times. Relative IFN-α expression then dropped down approximatively 10 times after 16 h of CpG stimulation, in comparison with the level of expression after 4 h. In pDCs purified from different donors HCV up-regulated IFN-α expression from 1 to 44 times.

Since the ability of CpG-A to induce IFN-α was severely impaired in the presence of HCVcc, we examined TLR7 and TLR9 expression in CpG-A-stimulated cells. Incubation of pDCs with CpG-A resulted in up-regulation of TLR7 expression in pDCs after 2 h and later. By contrast, expression of TLR9 was down-regulated. Priming of pDCs with HCVcc slowed down the TLR7 up- and TLR9 down-regulation by approximately 50%. Because triggering of TLR9 and/or TLR7 is known to activate the IRF-7-mediated pathway, we also examined the expression of this molecule, which is responsible for the transcription of IFN-α, and which is constitutively present in pDCs (Figure 4C). Modest up-regulation (1.6 times) of IRF-7 expression in CpG-A-stimulated pDCs was reduced by approximatively 50% in HCVcc-primed cells. Taken together, blockade of stimulation *via* TLR9 occurs early after exposure of pDCs to HCV and correlates with down-regulation of IRF7.

### HCVcc and HCV-LPs but not HCV core or envelope glycoprotein E2 inhibit pDC-associated production of IFN-α stimulated via TLR9

**Figure 5:**
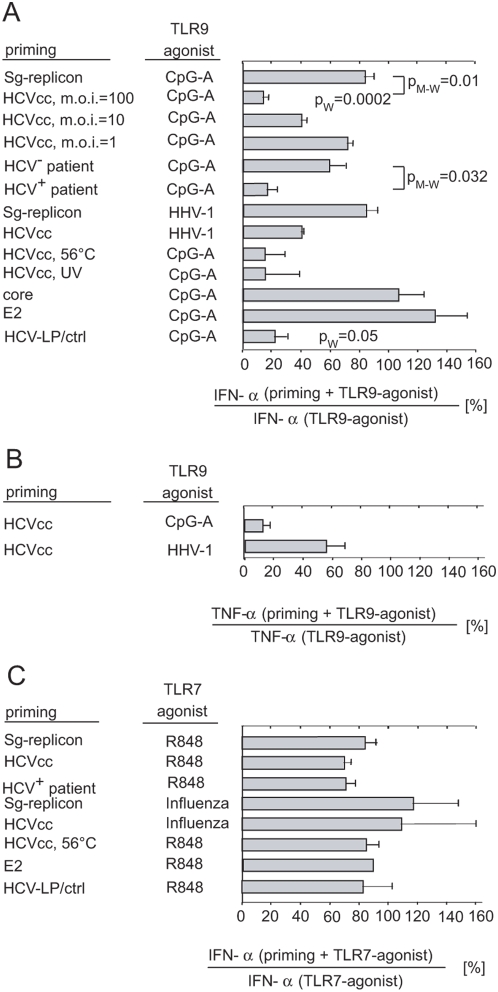
Effect of HCV, HCV-LPs, HCV core, and envelope glycoprotein E2 on the secretion of IFN-α from pDCs stimulated with TLR7 or TLR9 agonists. (A) Purified pDCs were exposed to 100, 10, or 1 HCVcc RNA copies per cell or to patient sera-derived HCV. Alternatively, pDCs were primed with the same quantity of virus inactivated with heat-treatment at 56°C for 30 min (HCVcc, 56°C) or with UV-treatment at 0.2 J/cm^2^ (HCVcc, UV), or exposed to noninfectious insect cell-derived HCV-LPs (0.1 µg E2/ml corresponding to approximately 5,000 particles per cell) or to control (ctrl) cell preparations (1 µg/ml). pDC were also incubated with HCV core or envelope glycoprotein E2 (10 µg/ml), or with an equivalent volume of control supernatant collected 16 h after UV irradiation of Huh7.5 cells transfected with HCV subgenomic replicon at 30 mJ/cm^2^ prepared in the same way as the viral stock (Sg-replicon, UV). pDCs were also primed with HCV virus particles (100 HCV genomes per cell) prepared from five different sera of chronically infected patients (HCV^+^ serum) or from equivalent volumes of four different sera of healthy individuals (HCV^−^ serum). Two hours later, primed or mock-primed pDCs were stimulated (A) with TLR9-agonists, with CpG-A (2.5 µg/ml), or with HHV-1 KOS (multiplicity of infection = 100), or (C) with TLR7-agonists, with resiquimod (R848, 0.5-µM), or with influenza virus A/H3N2/Johannesburg (multiplicity of infection = 100). Secretion of IFN-α (A, C) or TNF-α (B) in cell-free supernatant of pDCs was determined by means of ELISA analysis 1 day post-stimulation. The results are expressed as percentages of IFN-α production from pDCs that were first primed as specified above and then further treated with the respective TLR agonist relative to IFN-α production from pDCs stimulated only with the respective TLR agonist. p_W_, Wilcoxon matched pairs test used to compare differences between the distributions of IFN-α production in primed and non-primed pDC-cultures; p_M-W_, Mann-Whitney two-tailed non-parametric test used to compare differences between the distributions of IFN-α production in HCV-exposed and Sg-replicon-exposed pDC cultures, or in HCV^+^serum-exposed and HCV^−^ serum-exposed pDC cultures.

We assayed whether non-infectious HCV-LPs, HCV core (BAA01000), and envelope glycoprotein E2 disrupt TLR9-mediated production of IFN-α, in addition to the reported effect of HCVcc [23]). pDCs primed with HCVcc for 2 h and subsequently stimulated with CpG-A produced IFN-α at 11.2±5.4% of the levels produced by pDCs stimulated only with CpG-A (Figure 5A). This reduction was highly significant relative to that in CpG-A-treated cells from 10 healthy donors in 14 experiments (*p* = 0.0002) and relative to control supernatants from Huh7.5 cells transfected with subgenomic replicon and rendered apoptotic by UV irradiation (p = 0.01). Inhibition of TLR9-mediated production of IFN-α was HCVcc dose-dependent and dropped to negligible level in pDCs exposed in average to one HCVcc RNA molecule per cell.

To test the biological relevance of results obtained with cell culture-derived HCV, we determined the level of inhibition of TLR9-mediated activation by patient serum-derived HCV (Figure 5A). pDCs primed for 2 h with HCV from patients (n = 5) and subsequently stimulated with CpG-A produced IFN-α at 19.3±5.9% of the levels produced by pDCs stimulated only with CpG-A, whereas pDCs primed with sediments prepared by ultracentrifugation through a cushion of 20% sucrose of sera obtained from four HCV-negative individuals produced IFN-α at 58.3±11.6% of the levels produced by pDCs stimulated only with CpG-A (Figure 5A). The difference between inhibition of TLR9-mediated activation by HCV from patients' serum and healthy controls was significant (p = 0.032).

In addition to CpG-A, we tested the effect of HCV on the stimulation of pDCs by HHV-1, a naturally occurring TLR9 agonist (Figure 5A). Exposure of pDCs to HCV followed by inoculation with HHV-1 reduced IFN-α secretion by >60% in comparison with pDC culture exposed only to HHV-1. Control HCV virion-free supernatant from Huh7.5 cells transfected with HCV subgenomic replicons did not significantly block HHV-1-induced IFN-α secretion.

To determine whether replication-competent HCV is required for the inhibitory effect, pDCs were exposed to heat-inactivated or UV-inactivated virus or to HCV-LPs in the presence of CpG-A. Heat-inactivated as well as UV-inactivated HCVcc suppressed CpG-A-induced IFN-α secretion by >80%, as did the replication-competent virus (Figure 5A).

To further define the viral factors involved in this mechanism, we primed pDCs with non infectious HCV-LPs instead of HCV (Figure 5A). HCV-LPs are generated by self assembly of the HCV structural proteins in insect cells and have been shown to have similar biophysical, antigenic and immunogenic properties as HCV particles [25], [32]–[34]. Due to the lack of a functional genome and the nonstructural proteins, HCV-LPs are noninfectious. Since DCs efficiently take up, process and present HCV-LPs to HCV-specific CD4^+^ and CD8^+^ T cells, the interaction of HCV-LPs with DCs has allowed to identify mechanisms of HCV uptake and cross-presentation by human dendritic cells [25], [26]. Priming with HCV-LPs reduced the CpG-A–induced IFN-α levels by >75% compared to priming with control preparations. The absent inhibition of CpG-A–induced IFN-α production by control cell preparations produced from insect cells infected with a control baculovirus ruled out that contaminating cellular proteins of virus preparations were responsible for blocking TLR9 function. In contrast to HCVcc and HCV-LPs, HCV E2 (AAB30986) or core protein did not inhibit TLR9-mediated activation of pDCs. In conclusion, these data indicate that HCV virions or virus-like particles, but not the soluble forms of core and envelope glycoprotein E2 suppress stimulation *via* TLR9.

In addition to production of large amounts of IFN-α, TLR activation of pDCs can induce the production of proinflammatory cytokines such as TNF-α and IL-6. Similar to the marked inhibition of TLR9-mediated induction of IFN-α, exposure of pDCs with cell culture-derived HCV resulted in a more than 80% inhibition of CpG-A-induced TNF-α production (Figure 5B). A less pronounced effect of HCV on TNF-α production (blockade >40%) was observed when the cells were stimulated by HHV-1, a naturally occurring TLR9 agonist, instead of CpG-A (Figure 5B).

In marked contrast, HCV exerted no discernible effect on pDC response to TLR7-mediated induction of IFN-α secretion (Figure 5C). Neither HCV (12 experiments with pDCs from nine healthy donors), nor heat-inactivated HCV, nor HCV E2, nor patient-derived HCV, nor HCV-LPs, nor cell-free supernatant from Huh7.5 cells transfected with subgenomic replicon suppressed pDC-associated secretion of IFN-α induced with a synthetic ligand (resiquimod) or with a natural agonist (influenza virus) (Figure 5C). Thus, the HCV-induced suppression of TLR9-mediated IFN-α secretion is not due to a generalized effect of HCV on TLR signaling in pDCs.

## Discussion

In the present study, we demonstrate that HCV, in contrast to influenza virus or HHV-1, is a weak inducer of IFN-α in purified pDCs from healthy donors, and it does not induce pDC maturation. As in our study, no maturation was detected in pDCs from HCV-infected individuals [21], [23]. Sensitivity of IFN-α production to inhibitors of endocytosis (dimethyl amiloride, cytochalasin D, and chlorpromazine) suggests that HCV enters pDCs by the endocytosis pathway. Because TLR7- and TLR9-mediated signaling depends on acidification and maturation of endosomes [35]–[38], the endocytosis inhibitors that prevent endosomal acidification (chloroquine, quinarcine, bafilomycin A1 and NH_4_Cl) block IFN-α production but are not specific for endocytosis of HCV. A similar production of IFN-α induced by replication-competent virus or by viruses inactivated by heat or by UV-irradiation suggests that an HCV replication-competent phenotype is not required for pDC-associated production of IFN-α and is compatible with HCV recognition in pDCs by endocytosis mechanism. In hepatocytes, HCV enters target cells by clathrin-mediated endocytosis, followed by a fusion step from within an early acidic endosomal compartment [39], [40]. In contrast to hepatocytes, endocytosis of HCV in pDCs is not followed by expression of HCV genome and viral replication [21].

Differences in the number of assayed donors, in the viral strain, and virus titer could be the reason why no production of IFN-α was detected in *in vitro* HCV-exposed in some other studies [21], [23]. Limited production of IFN-α as well as the absence of pDC differentiation may contribute to the reduced innate and adaptive immune responses against HCV observed in the course of chronic infection [41]–[45]. Both HCV and HIV-1, which are related to chronic diseases accompanied by sustained plasma viremia, are weak inducers of IFN-α and TNF-α. In contrast, influenza virus and HHV-1, the potent inducers of IFN-α and TNF-α are related to diseases with transient viremia.

In order to better understand the low production of IFN-α by HCV-exposed pDCs, we studied HCV-induced blockage of TLR9-mediated production of IFN-α. We demonstrate that in addition to HCVcc [23], HCV-LPs also block TLR9-mediated production of IFN-α. It has recently been demonstrated that pDCs cultured in the presence of IL3 bind HCV-LPs [25]. Blockage of CpG-A-induced production of IFN-α by HCV-LPs unequivocally shows that replication-competent phenotype and expression of the nonstructural proteins of HCV are not required for the inhibition. Furthermore, these data demonstrate that an interaction of the viral particle with host cell factors during viral uptake and endocytosis is involved in inhibitory mechanism. Since recombinant soluble core and E2 proteins did not inhibit CpG-A induced production of IFN-α, it is likely that the presence of conformation-dependent epitopes within the HCV particle are required for HCV sensing by pDCs. It is possible that soluble forms of core and E2 proteins could be found in different compartments to HCV particles and could therefore be unavailable for TLR9-mediated inhibition of IFN-α secretion. Therefore, core and E2 proteins cannot be excluded from inhibitory effects. Alternatively, the E1 protein (which was not tested in this study) could play a role in mediating the inhibition to CpG-A induced production of IFN-α. A recent report by Amjad *et al.* [46] showed that non-structural proteins of HCV (namely NS3 and NS5) inhibit TLR9-induced IFN-α secretion. Because HCV particles do not contain non-structural proteins and because HCV does not express its genome in pDCs [21], it is difficult to interpret these results in the context of interaction of NS3 and NS5 with circulating pDCs studied in our work. However, this inhibition could be more important in infected liver, where the interaction of pDCs with NS3 and NS5 released from hepatocytes probably occurs. Further studies are underway to fine map the factors within the viral particle and to identify the host cell proteins mediating this effect.

Several mechanisms could be responsible for the weak responsiveness of pDCs to HCV on one hand, and the blockage of TLR9-mediated production of IFN-α on the other. It is conceivable that the poor secretion of IFN-α by pDCs could be related to HCV cross-linkage of a variety of cell surface receptors that down–regulate IFN-α production, such as BDCA-2 (Q8WTT0) [47], [48], DCIR (NP 919429) [49], ILT7-FcεRI gamma (AAD02203) [50], and FcεRI (CAA46955) [51]. Among them, ligation of DCIR is followed by co-localization of DCIR and CpG-A in endosomes, which results in a specific TLR9- but not TLR7-mediated inhibition of IFN-α and TNF-α [49]. Inhibition of both IFN-α and TNF-α was seen also in our experiments with HCVcc (Fig. 5A,B). It is conceivable that HCVcc and HCV-LPs are, after ligation of a cell surface receptor, transported to the vicinity of TLR9 and that they use (as a “wrong cargo”) the mechanism of spatiotemporal regulation of IFN-α induction [52] to escape from recognition by TLR7, the presumed natural receptor for HCV [53].

The blockade of TLR9-mediated production of IFN-α, effective only when pDCs were exposed to virus prior to or shortly after CpG-A stimulation, was already detectable at the IFN-α transcription level 2 h after stimulation with CpG-A and correlated with down-regulation of IRF7 expression. Whereas TLR7 and TLR9 signal transduction pathways downstream of Toll-IL-1R overlap, the regulation of the gene expression of TLR7 and that of TLR9 are substantially different. Up-regulation of TLR7 expression and down-regulation of TLR9 expression in CpG-A-stimulated pDCs, observed also in previous studies [54], [55], had been reduced to approximately 50% after pre-stimulation with HCV JFH-1. Constitutively lower expression levels of TLR9 compared to TLR7 in normal pDCs [56], [57] could enhance the former mechanisms and make TLR9 more vulnerable to inhibitory effects. Because pDCs stimulated through TLR9 are refractive to re-stimulation [58], we suppose that host cell proteins potentially involved in both traffic of the viral particle and inhibition of TLR signaling, make pDCs non-responsive to the second signal given by CpG-A. Taken together, rapidly and early occurring HCV particle-host cell protein interaction during particle internalization and endocytosis is followed by blockade of TLR9 by cellular host protein with impaired production of IFN-α. Supposed sequestration of viral particle in the proximity of TLR9 could result in less efficient sensing of HCV RNA by distal TLR7, without affecting TLR7 function, as shown by responses to resiquimod. Triggering the endocytosis of host molecule(s) that inhibit TLR9 signaling and transport virus particles toward TLR9, out of contact with TLR7, could represent a new mechanism by which HCV evades the immune system.

In spite of our efforts to minimize the presence of bystander activation factors in viral stocks, such as preparations of HCV JFH-1, it is possible that the virus preparations were contaminated with membranous vesicles and other RNA- and DNA-containing cellular components that were co-purified with the virus. This cellular material could theoretically participate, in addition to HCV, in the stimulation of pDC-associated production of IFN-α and in the suppression of CpG-A-induced IFN-α secretion from pDCs exposed to HCV. To address this issue, we stimulated pDCs with a suspension prepared from cell-free supernatant of apoptotic Huh7.5 cells transfected with HCV subgenomic replicon. This control HCV-free supernatant did not induce pDC-associated production of IFN-α and did not block CpG-A-induced IFN-α secretion. Furthermore, similar levels of IFN-α secreted from pDCs stimulated by HCV virions purified from different biological materials—recombinant cell culture-derived as well as patient-derived HCV—further confirm the induction of pDC-associated IFN-α by HCV and not by cellular components. Side-by-side control experiments using preparations of cell lysates containing all proteins or cellular factors potentially contaminating particle preparations confirm that HCV-LPs, and not contaminating material, blocked CpG-mediated activation of pDCs.

Variability of the levels of IFN-α produced by the HCV-exposed pDCs from different donors could reflect polymorphism of the HCV-induced inhibitory mechanisms and could result in different outcomes of HCV infection (spontaneous resolution versus chronicity). Optimal viral concentration that blocked CpG-A-mediated production of IFN-α by isolated pDCs corresponded to 10^8^ HCVcc RNA molecules (and approximately to 5×10^9^ HCV-LP particles) per milliliter. Only marginal inhibition was observed at 100 times lower HCVcc RNA concentration. Given that most chronically infected patients have levels of HCV RNA between 10^5^ and 10^7^ copies per milliliter, the virus concentration necessary for *in vitro* inhibition of TLR9-mediated production of IFN-α is compatible with the block of IFN-α in approximately 0.1 to 10% of circulating pDCs, and with the observation that individuals chronically infected with HCV are not immunocompromised.

Similar to our results obtained for HCV, several other viruses have been shown to block stimulation *via* TLR9. As recently shown by Fauci and colleagues [59], HIV-1 gp120 (AAC37925) - a BDCA-2 [59] and DCIR ligand [60] - inhibits TLR9-mediated activation and IFN-α secretion, but not TLR7-mediated activation and IFN-α secretion in pDCs. Hepatitis B virions also selectively inhibit TLR9-mediated activation and IFN-α secretion (I. E. Vincent, C. Trepo, personal communication [61]), showing that two hepatitis viruses impair the same pDC function.

Obvious caveats must be considered in transposition of *in vitro* results based on analysis of isolated pDCs exposed to HCVcc in an “acute setting” [23], to *ex vivo* experiments based of analysis of complex interactions of pDCs with monocytes/macrophages [19], [62] and NK cells [63] in “chronical setting”, and to pathogenesis of HCV in infected individuals. A better understanding of the stimulation of TLR7 and TLR9 with their synthetic ligands in the presence of HCV may identify new approaches for the development of antiviral strategies based on TLR agonists. Recent clinical studies have shown that administration of TLR7 agonists resiquimod [64] and isatoribine [65], as well as of TLR9 agonist CpG-A [66] results in reduction of plasma virus concentration in patients with chronic HCV infection.

## Materials and Methods

### pDC isolation and culture

We prepared peripheral blood mononuclear cells (PBMCs) using density gradient centrifugation on Lymphoprep (AbCys S.A., Paris, France). pDCs were purified directly from PBMCs by use of magnetic bead isolation kits followed by separation on AutoMacs (Miltenyi Biotech). The BDCA-4 diamond isolation kit (Miltenyi Biotech) was used and yielded levels of purity from 75% to 95%, with a contamination of less than 5% myeloid dendritic cells. In some experiments in which we wanted to achieve the highest purity of pDCs, we pre-enriched dendritic cells from PBMCs to 50–70% purity by means of magnetic bead depletion of erythrocytes, monocytes, and T-, B-, and NK-cells using MAbs against CD3-CD19-CD56-CD14-CD34-CD16-CD66b and glycophorin A (Human Dendritic Enrichment Kit: Stem Cell Technologies, Inc., Grenoble, France). We then separated pDCs from the enriched population by means of fluorescence-activated cell sorting (FACS-ARIA, Becton-Dickinson Bioscience, Erembodegem, Belgium) using FITC-conjugated lineage cocktail (CD3-CD14-CD216-CD19-CD20-CD56), PE-Cy5-conjugated CD123 MAb, Pe-Cy7-conjugated HLA-DR MAb, and APC-conjugated CD11C MAb. All conjugated MAbs were purchased from Becton-Dickinson. pDCs were gated as lin-FITC negative, HLA-DR-Pe-Cy7 positive, CD123-PE-Cy5 positive, and CD11c-APC negative. Levels of purity from 95% to 99% were repeatedly obtained. Isolated pDCs were cultivated in RPMI 1640 supplemented with 10% fetal calf serum and antibiotics. To optimize viability, recombinant IL3 (R&D Systems Europe, Ltd, Abingdon, UK) was added to a final concentration of 10 ng/ml.

### *In vitro* pDC stimulation

Purified pDCs were kept at the concentration of 10^6^ cells/ml in the medium containing IL3, for 2–3 h, aliquoted in 100-µl quantities in 96-well round-bottom culture plates, and stimulated in a final volume of 200 µl with medium alone, with CpG-A (ODN 2216 Invivogen), with CpG-A control, with resiquimod (a generous gift of 3M Pharmaceuticals, St. Paul, MN, USA), or with assayed viruses. Time curves performed revealed that an overnight incubation was optimal for quantifying the dendritic cell-associated cytokine production.

### Production and purification of cell culture-derived HCV (JFH1)

The HCV genotype 2A clone JFH-1, derived from a Japanese patient with fulminant hepatitis [67], [68], was prepared as previously described in detail [67]. Briefly, plasmid pJFH1 (kindly provided by T. Wakita, Tokyo Metropolitan Institute for Neuroscience, Tokyo, Japan) was used as a template for *in vitro* transcription with the MEGAscript™ T7 kit (Ambion, Austin, TX, USA). We then electroporated HCV RNA (10 µg) in 5×10^6^ Huh7.5.1 cells (kindly provided by S. L. Wieland and F. V. Chisari, The Scripps Research Institute, La Jolla, CA) at 270 V and 960 µF using a Bio-Rad Gene Pulser system. Transfected cells were then transferred to complete DMEM supplemented with 10% fetal calf serum and passaged every 3–5 days. The infectious endpoint titers of HCVcc were determined from production of cytopathic effect after infection of Huh7.5.1 cells with tenfold dilutions of virus-containing cell-free supernatant in duplicate. We prepared virus stocks by infecting 10^7^ Huh7.5.1 cells with 10^3^ TCID_Huh7.5.1_ of JFH-1 virus harvested from an RNA transfection experiment. Maximum titers of up to 10^5^ TCID_ Huh7.5.1_/ml in the supernatant were reached between 14 and 20 days post-infection. The titers of HCVcc genome-containing virus particles determined routinely with semiquantitative RT-PCR were 100 to 1,000 times higher than infectious titers. After 1,000-fold concentration by ultracentrifugation, the infectious titers typically increased 20-fold, whereas the titers of HCV genome-containing virus particles increased approximately 200-fold.

As a control, we used cell-free supernatant from Huh7.5 cells transfected with H/SG-neo (L+I) subgenomic replicon [30] (kindly provided by C. M. Rice, The Rockefeller University, New York, NY) UV-irradiated at 30 mJ/cm^2^ in a UV Stratalinker 1800 equipped with an integral UV photometer (Stratagene, La Jolla, CA).

### Patient-derived HCV

Each patient provided informed consent to participation in this study in accordance with institutional and regulatory guidelines. We quantified and genotyped HCV RNA from each serum sample using a branched DNA assay (Quantiplex HCV RNA 2.0 assay; Chiron Diagnostics) and a line probe assay (Inno-LiPA HCVII; Innogenetics), respectively. All serum samples were stored at −80°C until use.

### Production and purification of HCV-LPs

HCV-LPs derived from the cDNA of the infectious clone H77 were expressed and purified as described previously [24] [26]. Control preparations were derived from insect cells infected with a recombinant baculovirus containing the cDNA for β-glucuronidase (GUS) [24], [26]. The quantity of HCV-LPs was determined by analysis of the HCV-LP E2 concentration using an E2-specific ELISA [69] [26]. An HCV-LP E2 concentration of 0.1 µg/ml corresponded to approximately 5×10^8^ virus particles/100 µl or about 5,000 viral particles per cell (estimation of particles according to [32]).

### HIV-1, HHV-1, and influenza virus stocks

HIV-1 LAI virus stocks were prepared in PHA-activated PBMCs cultivated in RPMI 1640 medium supplemented with 200-U/ml recombinant IL-2 (Chiron), 15% fetal calf serum, and antibiotics as previously described in detail [70]. Stocks of HHV-1, strain KOS (ATCC, VR-1493), were produced in Vero cells. Influenza virus A/H3N2/Johannesburg/34/99 (kindly provided by M. Mehtali and A. Leon, Vivalis SA, Nantes, France) was produced in avian cell line EB14 clone 074.

### Virus concentration

Pooled supernatants from infected cells were centrifuged at 4,000 rpm for 7 min to remove cellular debris, passed through a Millex®-HV PVDF 0.45-µm filter (Millipore, Bedford, MA), and then pelleted at 40,000 rpm in a Beckman 45Ti rotor for 1 h. The virus pellets were resuspended in RPMI 1640 medium and centrifuged through a cushion of 20% sucrose at 35,000 rpm in a Beckman SW41 rotor for 2 h. The ultracentrifuged virus was resuspended in RPMI 1640 medium and centrifuged again at 35,000 rpm in the Beckman SW41 rotor for 2 h to remove the rest of the sucrose and to obtain a 1,000-fold concentrated virus suspension. HCV viral particles from the serum samples of infected patients and supernatants from Huh7.5 cells transfected with H/SG-neo (L+I) subgenomic replicon were ultracentrifuged and purified under the same conditions.

### HCVcc virus inactivation

Concentrated virus at 10^9^ RNA copies/ml was inactivated by heat treatment for 30 min at 56°C or by exposure to 0.2-J/cm^2^ UV with a UV Stratalinker 1800 equipped with an integral UV photometer (Stratagene, La Jolla, CA). pDCs were inoculated with virus in a quantity equivalent to 100 genome-containing virus particles per cell. This quantity of genome equivalents corresponded to an HCV infectious dose of 0.01–0.1 tissue culture infectious doses per Huh7.5.1 indicator cell.

### Quantitation of viral genome copies

Using the QIAamp viral RNA kit (Qiagen, Hilden, Germany), we isolated RNA from virions present in the ultracentrifuged virus. We determined endpoint dilution titers of viral genome copies with semiquantitative RT-PCR using the Superscript One Step RT-PCR system (Invitrogen, Cergy Pontoise, France). The HCV 5′-untranslated region was amplified by means of nested PCR as described previously [71]. Alternatively, HCV RNA was quantified on the basis of a real-time reverse transcription polymerase chain reaction using the primer RTU1 [71] for cDNA synthesis, primer pair UTR2 and RTU2 [71] for PCR amplification, and HCV JFH-1 RNA prepared *in vitro* by means of T7 polymerase as a standard.

For PCR amplification of the HIV LTR, we used the following primer pairs: 5′-CTGTGGATCTACCACACACAAGGCTAC (sense; L2) and 5′- GCTGCTTATATGTAGCATCTGAGGGC (antisense; L3) for the first round, and 5′-GATTGGCAGAACTACACACCAG (sense; L4) and 5′-CCAGCGGAAAGTCCCTTGTAG (antisense; L5) for the nested round.

Influenza virus Johannesburg H3N3 was amplified as described previously [72]. DNA from purified virions of HHV-1 (strain KOS) was extracted with SDS and proteinase K, purified further with phenol/chloroform, and amplified by means of PCR as described in [73]. Reaction mixtures were amplified in a Biometra T3 Thermocycler (Biometra, Goettingen, Germany) for 40 cycles at 95°C for 1 min, 60°C for 1 min, and 72°C for 1 min.

### Gene expression assay and analysis

We extracted total RNA using the Qiagen RNeasy micro kit (Qiagen, Courtaboef, France) and converted it to cDNA using random hexamers (High Capacity cDNA RT kit, Applied Biosystems, Courtaboef, France). We assayed human IFN-α, IRF7, TLR7, and TLR9 using Taqman Gene Expression Assay primers with labeled probes (Applied BioSystems). Threshold cycle (C_T_) values for each gene were normalized to GAPDH. The negative control for each experiment, stimulation of pDCs with medium alone, was assigned a value of 1, and all data are expressed as fold induction over the negative control.

### Immunofluorescence analysis

For analysis of cell surface marker expression, cells were incubated for 15 min at room temperature in the presence of FITC-conjugated CD80, FITC-conjugated CD83, PE-conjugated CD40, PE-conjugated CD86, PE-Cy5-conjugated CD123, and PE-Cy7-conjugated HLA-DR MAbs (all purchased from BD or Beckmann Coulter). pDC apoptosis was detected using FITC-conjugated Annexin V and 7-amino-actinomycin D (7ADD) in Annexin V-binding buffer (Becton-Dickinson). pDCs were gated based on size and granularity (FSC/SSC) and analyzed for the presence of fluorescent cells. After labeling, cells were fixed in 4% paraformaldehyde and analyzed after gating on live CD123^+^/HLA-DR^+^ cells with a FACS-ARIA using DIVA software (Becton-Dickinson, Le Pont de Claix, France). Data were analyzed by means of Flowjo software.

### Determination of IFN-α and TNF-α production by ELISA

To measure the quantities of total IFN-α and TNF-α produced, we collected supernatants from parallel cultures after 16 to 20 h and assayed them using a human IFN-α ELISA (PBL Laboratories) or a TNF-α ELISA (Opti-EIA set, Pharmingen), respectively.

### Endocytosis and endosomal maturation inhibitors

pDCs were cultured in the presence of 10^7^ HCV JFH-1 per 10^5^ cells per 200 µl of medium and of various endocytosis and endosomal maturation/acidification inhibitors: dimethyl amyloride (50 µM), cytochalasin D (10 µM), chlorpromazine (6.25 µg/ml), chloroquine (5 µM), quinacrine (5 µM), NH_4_Cl (1 mM), and bafilomycin A1 (50 nM), all from Sigma-Aldrich. Supernatants were tested after 20 h for the IFN-α produced.

### Statistical analysis

To compare the levels of IFN-α production by pDCs exposed to different viruses, we used a nonparametric Mann-Whitney test. To compare responses to TLR agonists in the presence versus absence of HCV, we used the Wilcoxon matched pairs test. Data were analyzed with Prism 4 Biostatistics software. All tests of significance were two-sided, and a *p* value ≤0.05 was considered to be significant.

## Supporting Information

Figure S1 Purity of viral preparations. (A) Concentrated supernatant from Huh7.5.1 cells infected with HCV JFH-1 or similarly concentrated supernatant from minireplicone transfected Huh7.5.1 cells collected 16 h after UV irradiation with a 30 mJ/cm2 dose were adsorbed onto collagen membrane-coated electron microscopy grids. The adsorbed materials were negatively stained with 1% uranyl acetate or with 1% sodium phosphotungstate and observed in a Zeiss MET-EM 912 microscope. The presence of virus-like particles of 60–80 nm in diameter in the negatively stained viral preparation is shown by arrows. (B) Viral like particles or exosomes are absent in supernatant from Huh7.5 cells transfected with subgenomic (Sg) replicons. (C) We determined the quantity and quality of RNA present in the viral preparation using the Agilent 2100 bioanalyzer and RNA LabChip® kit. Contamination of virus preparation with RNA material was below the detection limits of the control methods used in our experiments (≤1 ng of RNA/ml).To determine the level of contamination of viral preparations with cellular DNA, we also amplified by means of GAPDH-specific PCR the DNA molecules presumably present in 5-µl aliquots (5×10^6^ genome-containing virus particles) of viral stock used to stimulate pDC cultures. No GAPDH-specific signal was detected in 4 assayed aliquots (not shown).
(4.53 MB PDF)

Figure S2 Secretion of IFN-α induced with molecular clone HCV JFH-1 and with resiquimod in pDCs from different normal healthy donors. Cell cultures of pDCs purified from different normal healthy donors, adjusted to a concentration of 10^6^ cells/ml in the presence of IL-3, were inoculated with 100 HCV RNA-containing virus particles per cell or stimulated with resiquimod (R848, 0.5 µM) in a total volume of 200 µl. Secretion of IFN-α in cell-free supernatant was determined by means of ELISA analysis 1 day post-stimulation. Each point represents a different donor analyzed in Figure 1.
(0.49 MB PDF)
