# Supplementary figures and images for: Hepatitis C Virus Is a Weak Inducer of Interferon Alpha in Plasmacytoid Dendritic Cells in Comparison with Influenza and Human Herpesvirus Type-1
Source: PLoS One. 2009 Feb 2;4(2):e4319. doi: 10.1371/journal.pone.0004319 (PMC2629532; doi:10.1371/journal.pone.0004319)

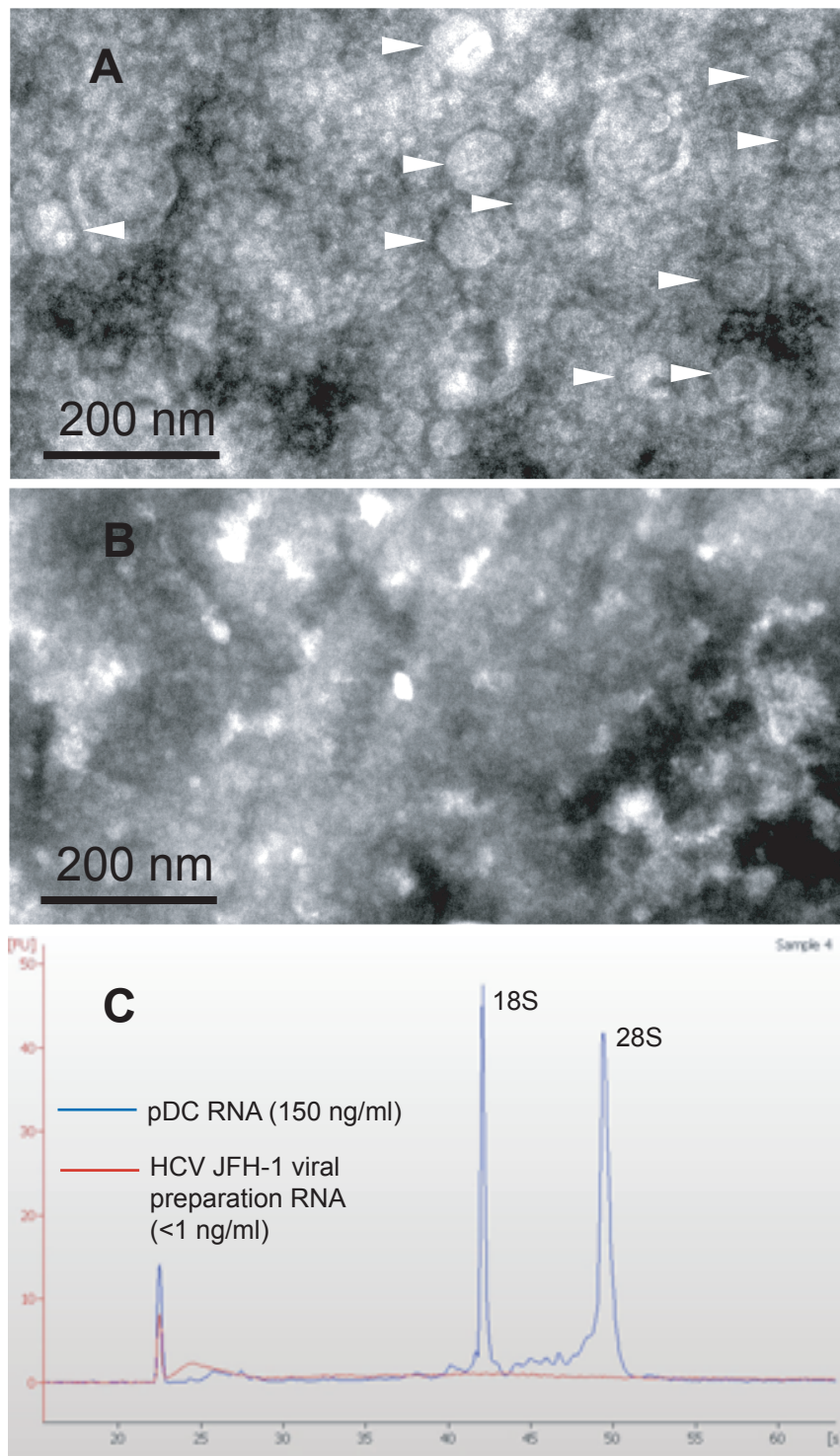

Figure S1

Supplement: Figure S1 — Purity of viral preparations. (A) Concentrated supernatant from Huh7.5.1 cells infected with HCV JFH-1 or similarly concentrated supernatant from minireplicone transfected Huh7.5.1 cells collected 16 h after UV irradiation with a 30 mJ/cm2 dose were adsorbed onto collagen membrane-coated electron microscopy grids. The adsorbed materials were negatively stained with 1% uranyl acetate or with 1% sodium phosphotungstate and observed in a Zeiss MET-EM 912 microscope. The presence of virus-like particles of 60–80 nm in diameter in the negatively stained viral preparation is shown by arrows. (B) Viral like particles or exosomes are absent in supernatant from Huh7.5 cells transfected with subgenomic (Sg) replicons. (C) We determined the quantity and quality of RNA present in the viral preparation using the Agilent 2100 bioanalyzer and RNA LabChip® kit. Contamination of virus preparation with RNA material was below the detection limits of the control methods used in our experiments (≤1 ng of RNA/ml).To determine the level of contamination of viral preparations with cellular DNA, we also amplified by means of GAPDH-specific PCR the DNA molecules presumably present in 5-µl aliquots (5×10^6^ genome-containing virus particles) of viral stock used to stimulate pDC cultures. No GAPDH-specific signal was detected in 4 assayed aliquots (not shown). (4.53 MB PDF) [file pone.0004319.s001.pdf]

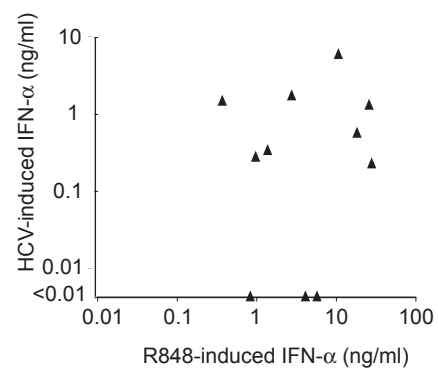

Figure S2

Supplement: Figure S2 — Secretion of IFN-α induced with molecular clone HCV JFH-1 and with resiquimod in pDCs from different normal healthy donors. Cell cultures of pDCs purified from different normal healthy donors, adjusted to a concentration of 106 cells/ml in the presence of IL-3, were inoculated with 100 HCV RNA-containing virus particles per cell or stimulated with resiquimod (R848, 0.5 µM) in a total volume of 200 µl. Secretion of IFN-α in cell-free supernatant was determined by means of ELISA analysis 1 day post-stimulation. Each point represents a different donor analyzed in Figure 1. (0.49 MB PDF) [file pone.0004319.s002.pdf]
